# A Prebiotic Formula Improves the Gastrointestinal Bacterial Flora in Toddlers

**DOI:** 10.1155/2016/3504282

**Published:** 2016-06-14

**Authors:** Ya-Ling Chen, Fang-Hsuean Liao, Shyh-Hsiang Lin, Yi-Wen Chien

**Affiliations:** ^1^Department of Nutrition and Health Sciences, Chang Gung University of Science and Technology, Taoyuan 333, Taiwan; ^2^School of Nutrition and Health Sciences, Taipei Medical University, Taipei 11014, Taiwan

## Abstract

We aimed to investigate the effect of enriched 3-prebiotic formula (including inulin, fructooligosaccharides, and galactooligosaccharides) on toddler gut health by measuring fecal microbiota. Our results revealed that the consumption of 3-prebiotic formula three times per day giving total intake of 1.8 g prebiotic ingredients significantly showed the increased number of probiotic* Bifidobacterium* spp. colonies and the reduced populations of both* C. perfringens* and total anaerobic bacteria on the fecal bacterial flora in toddlers at 18~36 months. In addition, total organic acids in the fecal samples significantly increased which improves the utilization of bifidus under acidic conditions after consumption of the 3-prebiotic formula. Therefore, using the formula enriched with prebiotic may maintain gut health in toddlers.

## 1. Introduction

The intestinal flora of newborn infants is an important physiologic factor in gut function and development of the immune system, which decreases as a child ages. Probiotics, such as lactobacilli and bifidobacteria, have been reported to increase populations of friendly bacteria and inhibit activities of harmful or pathogenic microbes in the human intestines and to be beneficial for maintaining individuals' health [1, 2]. Many studies have shown that infant formulas supplemented with probiotics, such as* Bifidobacterium* spp., can increase the population of bifidobacteria, enhance mucosal resistance against gastrointestinal infections, and reduce gut disorders [3–6].

Prebiotics are defined as indigestible food ingredients that are specifically fermented by bifidobacteria. Their presence in the intestines beneficially affects the host by selectively stimulating not only the growth and activity of bacteria and the formation of bacterial flora in the colon, but also the host's immune defenses, thus improving host health [7]. It was verified that inulin, lactulose, fructooligosaccharides, isomaltooligosaccharides, and galactooligosaccharides possess prebiotic properties [8–10]. Human milk oligosaccharides were reported to selectively stimulate the growth of bifidobacteria and lactobacilli in the intestines that may directly contribute to natural defenses against infection [11–13]. The composition and structure of human milk oligosaccharides cannot be reproduced by food processing; therefore, prebiotics are being considered for fortification of infant formulas. Cow milk-based infant formulas supplemented with a prebiotic mixture of galacto- and fructooligosaccharides can stimulate intestinal flora and the numbers of fecal bifidobacteria and lactobacilli similar to that of breast-fed infants [14, 15]. Inulin is composed of a group of fructose polymers that are only partially digested by upper-intestinal enzymes. Moreover, inulin was shown to consistently increase absorption and retention of several minerals and to improve bone mineralization [16–18].

The aim of the current study was to examine the effect of supplementing toddler formulas with three prebiotic ingredients (3-prebiotic formula), including inulin, fructooligosaccharides, and galactooligosaccharides, on the gastrointestinal bacteria flora and human physiology among 18~36-month-old children.

## 2. Materials and Methods

### 2.1. Subjects

Toddlers aged 1.5~3 years were screened by a pediatric physician. None had any evidence of gastrointestinal diseases or other health problems. For 2 weeks before the experiment, none of the subjects took any antibiotics or any drugs or supplements that might affect the gastrointestinal bacteria. A detailed explanation of the study design was given to the parents of subjects before participating in this trial. Each parent of subjects was enrolled with written informed consent. The study was approved by Taipei Medical University Research Ethics Committee.

### 2.2. Treatment

The toddler formula (3-prebiotic formula) was supplemented with three prebiotic ingredients, including inulin, fructooligosaccharides, and galactooligosaccharides (600 mg per 240 mL), provided by Bristol-Myers Squibb (Taipei, Taiwan).

### 2.3. Experimental Design and Process

We conducted an 8-week dietary intervention trial with 30 1.5~3-year-old children. The study period for each toddler was 8 weeks, comprising an initial 1 week of consuming formula without prebiotics (control period), an administration period of 6 weeks of consuming 240 mL of the 3-prebiotic formula three times a day, and 1 week of follow-up with the original formula without prebiotics.

During the experimental period, parents daily filled out a questionnaire on the fecal samples frequency and consistency. Consistency scores were recorded as follows: 1 = watery; 2 = soft and formed; 3 = hard. Fecal samples were collected and analyzed for the fecal bacterial flora on days 1, 7, 21, 35, 49, and 56. The fecal bacterial flora assessed was* Bifidobacterium* spp.,* Clostridium perfringens*, and total anaerobic bacteria. Organic acids, including lactate, acetate, propionate, and butyrate, were analyzed on days 7 and 49.

*Bifidobacterium* spp. in the fecal samples were used as an indicator of the probiotics in the intestinal tract and were incubated on bifidobacterium iodoacetate medium-25 (BIM-25), while* C. perfringens* was an indicator bacterial pathogen in the intestines and was incubated on tryptose-sulfite-D-cycloserine (TSC) agar with 100 mL of a D-cycloserine solution and 100 mL of 50% egg yolk emulsion. Total anaerobic bacteria in the feces were calculated on CDC Anaerobe Blood Agar plates and were used as the reference. Colonies were counted after 48 h of incubation to determine colony-forming units (cfu) per gram of wet weight of feces. The logarithm-values of the colonies of* Bifidobacterium* spp.,* C. perfringens*, and total anaerobic bacteria in each gram of feces represented the gastrointestinal bacterial flora of subjects.

Organic acids such as lactic acid, acetic acid, propionic acid, and butyric acid in the feces were analyzed with high-performance liquid chromatography (HPLC). Briefly, precisely 4 g of feces was mixed with 25 mL acetonitrile thoroughly before being centrifuged at 20,000 ×g for 30 min. The supernatant was removed and requantified to 25 mL before being syringe-filtered through a Titan (pore size: 0.2 *μ*m) filter. The final filtrate was injected into an HPLC system containing an Aminex HPX-87H column (300 × 7.8 mm), a solution of 3.75 mM sulfuric acid as the mobile phase, and a UV. The flow rate was set at 0.4 mL/min and the column temperature was set at 65°C.

### 2.4. Statistical Analysis

Data are expressed as the mean ± SEM. A paired *t*-test was performed on each of the variables to assess mean differences across time with SPSS software (Chicago, IL, USA). Differences were considered significant for *p* values of <0.05.

## 3. Results

### 3.1. Physiological Effect of Supplementation with the 3-Prebiotic Formula

Overall, 38 subjects (17 boys and 21 girls) completed the entire experiment; three subjects withdrew from the study due to personal reasons. Baseline characteristics are shown in Table 1. The frequency of diarrhea among subjects significantly decreased between weeks 0 and 6 (*p* < 0.05) of the administration period according to descriptions in the parents' questionnaires (data not shown).

### 3.2. Effect of 3-Prebiotic Formula Administration on the Fecal Bacteria Flora

After ingestion of the 3-prebiotic formula, the number of* Bifidobacterium* spp. colonies significantly increased compared to that at week 0 (*p* < 0.05) and was significantly lower after the follow-up period (Table 2). Conversely, the population of* C. perfringens* significantly decreased between weeks 0 and 6 of the administration period, and the increase after the follow-up period was notably lower than that in the control period and at the baseline. A decrease with a time-response effect was noted in the number of* C. perfringens* colonies after subjects consumed the 3-prebiotic formula. During the administration period, total anaerobic bacteria were significantly reduced with time (*p* < 0.05), although total anaerobic bacteria in the follow-up period were lower than those in the control period (*p* < 0.05).

**Figure 1:**
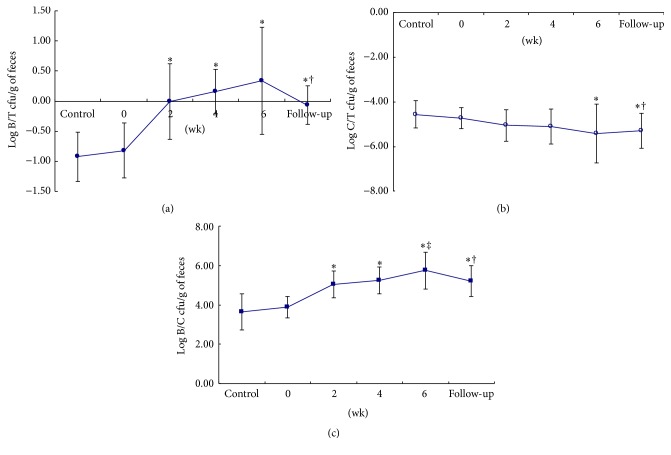
Effect of 3-prebiotic formula administration on the ratio of fecal bacterial flora in healthy subjects. Numbers of* Bifidobacterium* spp. (B),* Clostridium perfringens* (C), and total anaerobic bacteria (T) are expressed as log values of colony-forming units (cfu) per gram weight of feces. (a) Log_10_ B/T cfu/g wet weight of feces. (b) Log_10_ C/T cfu/g weight of feces. (c) Log_10_ B/C cfu/g wet weight of feces. In the control period and follow-up period, the control formula was used and not the 3-prebiotic formula. ^*∗*^Values significantly differ compared to that of week 0 of the administration period (*p* < 0.05). ^†^Values significantly differ compared to that of the control period (*p* < 0.05). ^‡^Values significantly differ compared to that at week 2 of the administration period (*p* < 0.05).

We made further observations of the growth of friendly and harmful bacteria in the gastrointestinal tract after ingestion of the 3-prebiotic formula. The population of* Bifidobacterium* spp. was compared to the total anaerobic bacteria, and an increase was observed during the experimental period (*p* < 0.05), which significantly differed from that of week 0, although the ratio was found to have decreased in the follow-up period (*p* < 0.05) and was still significantly higher than that in the control period (Figure 1(a)). Compared to the ratio of* C. perfringens* to the total anaerobic bacteria in each phase, the ratio gradually decreased during the experimental period (*p* < 0.05), and the decrease remained until the end of the study (Figure 1(b)). Moreover, the ratio of* Bifidobacterium* spp. to* C. perfringens* during ingestion of the 3-prebiotic formula significantly increased with a time-response effect compared to week 0 of the administration period (*p* < 0.05). Additionally, the ratio at week 6 was even higher than that at week 0 of the administration period (*p* < 0.05). The ratio in the follow-up period was lower but still showed an increase compared to week 0 of the administration and control periods (*p* < 0.05, Figure 1(c)).

### 3.3. Effect of the 3-Prebiotic Formula Administration on Organic Acids

**Figure 2:**
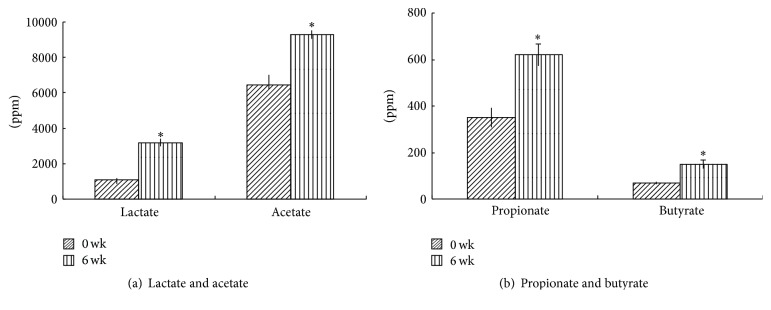
Effect of 3-prebiotic formula administration on contents of fecal organic acids. (a) Products of lactate and acetate and (b) propionate and butyrate. Data are expressed as the mean ± SD. ^*∗*^Values significantly differ compared to that of week 0 of the administration period (*p* < 0.05).

The index of organic acids, including lactate, acetate, propionate, and butyrate, significantly increased after the administration period compared to levels measured before the administration period (Figure 2).

## 4. Discussion

Probiotics such as bifidobacteria and lactobacilli are reported to prevent gastrointestinal diseases and disorders [19, 20]. We found that administration of the 3-prebiotic formula increased the growth of* Bifidobacterium* spp., decreased the growth of* C. perfringens*, and improved the host gastrointestinal bacterial flora, thus decreasing diarrhea conditions in toddlers. There are abundant bifidobacteria in the feces of healthy breast-fed infants and they have lower incidence of infantile diarrhea. As infants gradually grow up and develop into toddlers, there are many causes that induce children's diarrhea. The increase in* C. perfringens* bacteria is an abnormal condition of the colonic microbial population, and the decrease in* Bifidobacterium* spp. shows that the body is in an unhealthy state [21]. When populations of pathogenic bacteria that produce exotoxins or result in abnormal colonic microbial populations in the upper part of the intestines increase, diarrhea may occur. Recent reports indicated that supplementation with* Bifidobacterium* spp. and* Lactobacilli acidophilus* may enhance innate mucosal immune defense to protect against some immune-based disorders, inhibit the growth of intestinal pathogenic bacteria, lower the instances of diarrhea and bacterial infections induced by antibiotics or rotaviruses, and promote the health of the host [22–24]. Because enzymes in mammals cannot hydrolyze the *α*-1,6 linkages of carbohydrates, ingestion of oligosaccharides can increase* Bifidobacterium* spp. in the feces due to increases in probiotics in the intestines [25]. Similar to the results of the present study, supplementation with three probiotics, inulin, fructooligosaccharides, and galactooligosaccharides, selectively stimulated the indigenous bifidobacteria and lactobacilli and proliferation of bifidobacteria and lactobacilli after 4 weeks of ingestion of a 3-prebiotic formula. Moreover, we found that the ratio of* Bifidobacterium* spp. to total anaerobic bacteria and the ratio of* C. perfringens* to the total anaerobic bacteria improved compared to those before ingesting the 3-prebiotic formula. This indicates that the 3-prebiotic formula might improve the gastrointestinal bacterial flora of toddlers.

Oligosaccharides stimulate the growth of bifidobacteria and lactobacilli. One study showed that these bacteria may reduce the survival of pathogens by producing organic acids [26]. Organic acids, such as lactate, acetate, propionate, and butyrate, make up 85%~95% of short-chain fatty acids (SCFAs) in the large intestines. SCFAs provide energy to colonocytes and are the main fermentation products of the microbial breakdown of carbohydrates in the large intestine. We found that the 3-prebiotic formula significantly increased lactic acid and SCFA production. Some researchers indicated that the presence of butyrate regulates the differentiation of normal colonocytes [27] and increases the sensitivity of immune reactions to cancer cells [28]. It was also shown that butyrate inhibited risk factors for colon cancer and adenomas [29–31]. Furthermore, the production of lactic acid and SCFAs after ingestion of a 3-prebiotic formula may indirectly result in a more-acidic environment, which extensively inhibits most gram-positive and gram-negative bacteria [8, 21, 24, 32]. Under acidic conditions, the growth bacterial pathogen is suppressed, and the utilization of bifidus in an infant's intestines is enhanced [33].

Organic acids that induce decreases in intestinal pH values in the human gut tract may increase the absorption of some specific minerals, such as iron and calcium [34, 35]. Several studies have demonstrated that both inulin and oligosaccharides are effective prebiotics and also benefit the bioavailability of minerals including calcium and magnesium [36–38] and gut health [39, 40]. For adults, doses of prebiotics more than 8 g/day increased the absorption and retention of Ca [38]. Thus, the 3-prebiotic formula containing 250 mg/100 mL might have a similar positive effect on the intestinal tract of toddlers.

Overall, the 3-prebiotic formula increased the growth of probiotics and continued to produce more organic acids. By increasing the production of lactic, acetic, and propionic acids by bifidobacteria, a lower pH value may enhance the activity of bifidus and utilization of minerals.

## Figures and Tables

**Table 1:** Subject characteristics.

	Subjects
*N*	38
Boys (*n*)/girls (*n*)	17/21
Age (years)^1^	2 ± 0.6
Height (percentile, th)	75
Weight (percentile, th)	50~75

^1^Values are expressed as the mean ± SD.

**Table 2:** Effects of 3-prebiotic formula administration on the fecal bacterial flora.

	Control period (1 week)	Administration period (6 weeks)				Follow-up period (1 week)
		0	2	4	6	
B	9.1 ± 0.5	9.2 ± 0.4	9.5 ± 0.4^*∗*^	9.5 ± 0.2^*∗*^	9.6 ± 0.3^*∗*^	9.4 ± 0.2^†^
C	5.5 ± 0.7	5.3 ± 0.5	4.4 ± 0.5^*∗*^	4.3 ± 0.7^*∗*^	3.8 ± 0.9^*∗*^	4.2 ± 0.7^*∗*†^
T	10.0 ± 0.3	9.98 ± 0.2	9.5 ± 0.3^*∗*^	9.3 ± 0.3^*∗*^	9.2 ± 0.7^*∗*^	9.5 ± 0.3^*∗*†^

Data are expressed as colony-forming units (cfu) in log (numbers per gram of feces). B, *Bifidobacterium* spp.; C, *Clostridium perfringens*; T, total anaerobic bacteria.

^*∗*^Values significantly differ compared to that at week 0 of the administration period (*p* < 0.05). ^†^Values significantly differ compared to that of the control period (*p* < 0.05).
